# The Effect of Endometrial Thickness on Pregnancy, Maternal, and Perinatal Outcomes of Women in Fresh Cycles After IVF/ICSI: A Systematic Review and Meta-Analysis

**DOI:** 10.3389/fendo.2021.814648

**Published:** 2022-02-11

**Authors:** Zhiqi Liao, Chang Liu, Lei Cai, Lin Shen, Cong Sui, Hanwang Zhang, Kun Qian

**Affiliations:** ^1^ Reproductive Medicine Center, Tongji Hospital, Tongji Medical College, Huazhong University of Science and Technology, Wuhan, China; ^2^ Reproductive Medicine Center, The Affiliated Drum Tower Hospital of Nanjing University Medical College, Nanjing, China

**Keywords:** endometrium, *in vitro* fertilization, intracytoplasmic sperm injection, pregnancy rate, pregnancy complications

## Abstract

**Background:**

Thin endometrium on ovulation triggering day is associated with impaired pregnancy outcomes in women after *in vitro* fertilization/intracytoplasmic sperm injection (IVF/ICSI), but the role of thick endometrium on pregnancy outcomes remains controversial. Moreover, there has been insufficient evidence currently to analyze the influence of endometrial thickness (EMT) on obstetric complications and perinatal outcomes. Thus, we performed this meta-analysis to evaluate the effect of EMT on pregnancy, maternal, and perinatal outcomes in an enlarged sample size.

**Methods:**

The databases Pubmed, Embase, Cochrane Libraries, and Web of Science were searched for English articles evaluating the correlation between EMT and pregnancy, maternal, or perinatal outcomes in women who underwent IVF/ICSI. We included studies that depicted a clear definition of outcomes and EMT grouping on ovulation triggering day. The EMT effect was analyzed in fresh cycle. Qualities of studies were assessed by the Newcastle-Ottawa Scale (NOS). Odds ratios (ORs) and weighted mean difference (WMD) with 95% confidence intervals (CIs) were calculated for analyzing dichotomous and continuous outcomes respectively, under a fixed or random effect model.

**Results:**

A total of 22 pieces of literature were included for the final meta-analysis. A decreased trend towards pregnancy outcomes was observed, such as live birth rate (LBR), clinical pregnancy rate (CPR), and implantation rate (IR) in the thin endometrium groups (EMT <7 mm). In contrast, thick endometrium (EMT >14 mm) had no effect on pregnancy outcomes compared to medium EMT groups (EMT 7–14 mm). Moreover, thin endometrium (EMT <7.5 mm) enhanced the incidence of hypertensive disorders of pregnancy (HDP) and small-for-gestational-age (SGA) infants, and decreased the birthweight (BW) of babies.

**Conclusions:**

Our studies indicated that thin endometrium not only had detrimental effect on pregnancy outcomes, but also increased the risk of HDP in women and SGA of babies, or decreased BW of babies. The thick endometrium does not have an adverse effect on IVF outcomes. Therefore, patients need to be informed on possible obstetric complications and perinatal outcomes caused by thin endometrium and are encouraged to actively cooperate with perinatal care.

**Systematic Review Registration:**

(https://www.crd.york.ac.uk/PROSPERO/display_record.php?RecordID=242637), identifier CRD42021242637.

## Introduction

Assisted reproductive technology (ART), namely, *in vitro* fertilization (IVF) and intracytoplasmic sperm injection (ICSI), have been accepted as effective options for treating infertility (1). Multiple factors contribute to the success of IVF/ICSI, such as age, embryo quality and endometrial condition (2). Herein, endometrial thickness (EMT) measured by ultrasound has become a common indicator for monitoring endometrial condition, as the procedure of ultrasonographic examination is widely available and noninvasive (3). It has also been reported that EMT on ovulation triggering day was associated with the outcome of IVF/ICSI (4).

Many studies found that patients with thin endometrium had lower chances to be pregnant, both in fresh cycles and frozen-thawed embryo transfer (FET) cycles (5, 6). However, the relationship between increased EMT (>14 mm) and pregnancy outcomes remains controversial. Weissman et al. demonstrated that women with thick endometrium had lower implantation and pregnancy rate, and higher miscarriage rate (7). On the contrary, a study from Zhang et al. showed that increased EMT tended to improve IVF treatment outcomes, such as clinical pregnancy rate (CPR) (8). Therefore, there is lacking of consensus on the effect of thick endometrium on pregnancy outcomes of IVF/ICSI.

Furthermore, maternal perinatal complications and neonatal health are of great concern following ART as well (9, 10). Notably, recent evidence indicated that EMT has a strong correlation with maternal and perinatal outcomes (11–13). Guo et al. revealed that the incidence of small-for-gestational-age (SGA) infants was higher in thin endometrium group (13). Besides, Liu et al. also found that there was more risk of hypertensive disorders of pregnancy (HDP) in women with thin EMT (14). Nonetheless, the influence of thin EMT on obstetric complications and perinatal outcomes still lack evidence from a large sample size. Hence, this systematic review and meta-analysis aimed to assess the correlation between the EMT and pregnancy, maternal, and perinatal outcomes after IVF/ICSI.

## Methods

We performed this review according to the Preferred Reporting Items for Systematic Review and Meta-analysis (PRISMA) statement and a registered protocol (PROSPERO registration number: CRD42021242637).

### Search Strategy and Data Collection

We searched four databases, namely, Pubmed, Embase, Cochrane Libraries, and Web of Science, for studies about the association between the EMT and outcomes of IVF/ICSI with no country or article type restrictions. Articles that published in English until April 2021 were recruited. The following terms were used: [(*in vitro* fertilization) OR (intracytoplasmic sperm injection) OR (artificial reproductive technology)] AND [(endometrial thickness) OR (endometrial sonographic parameters) OR (endometrial characters) OR (endometrial receptive)] AND [(live birth rate) OR (pregnancy outcomes) OR (neonatal outcomes) OR (maternal outcomes) OR (obstetric outcomes) OR (treatment outcomes)] (**Supplementary Table 1**).

**Figure 1:**
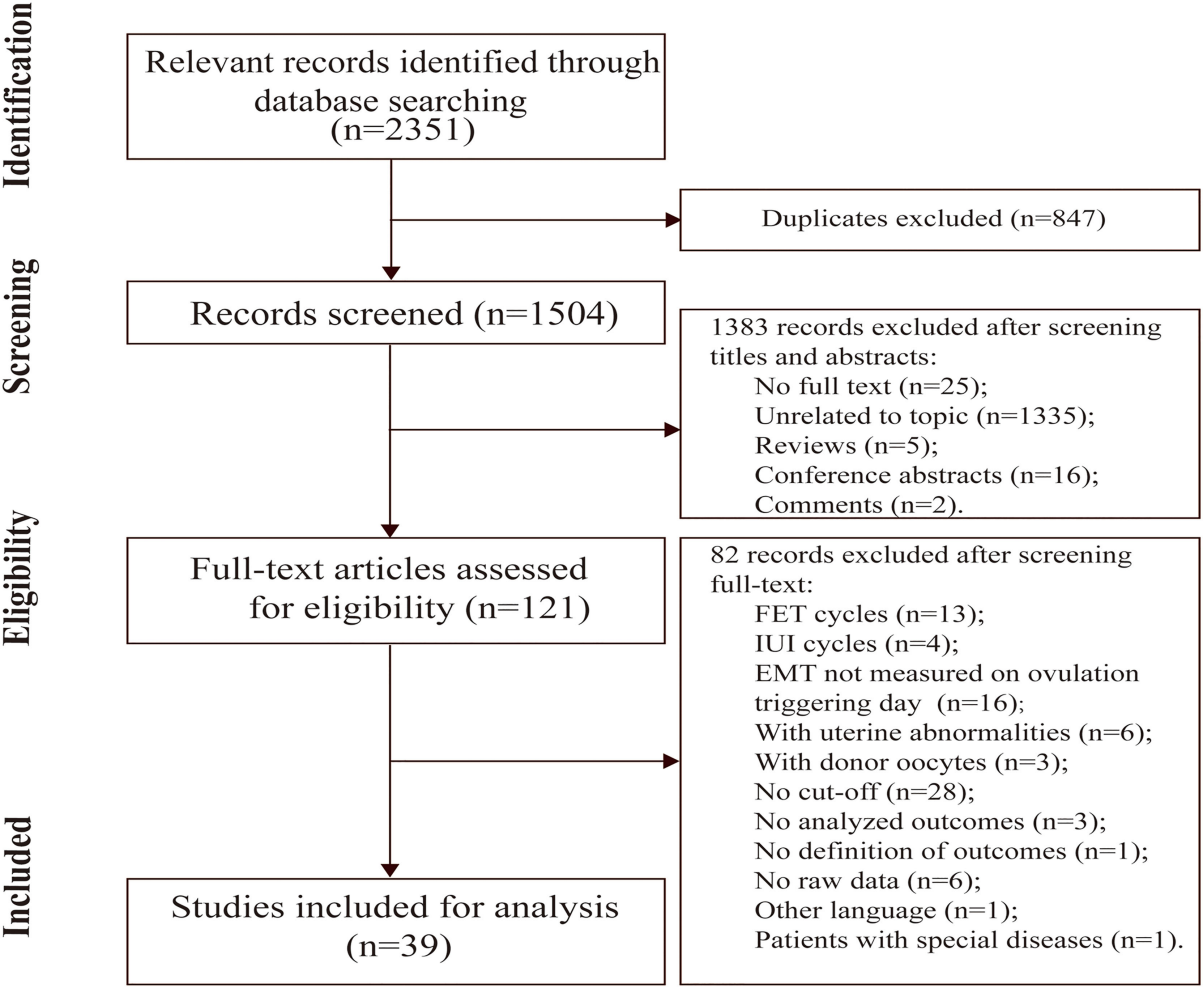
Flow chart of searching and screening strategy.

After excluding duplicates, titles and abstracts were screened by two independent reviewers (ZL and LC). Studies relevant to our topic were assessed for eligibility. The flow chart of search strategy is shown in **Figure 1**. Full-text articles that met inclusion criteria were reviewed, data of which were extracted and recorded in pre-designed spreadsheets by two authors independently (ZL and LC). Any disagreement was resolved *via* discussion or consulting the third author (CL) if the consensus could not be reached. The following data were collected: authors, published year, type of study, time period, country, the number of live birth and clinical pregnancy, the number of implantation and miscarriage, the occurrence of obstetric complications [i.e., placenta previa (PP), placenta abruption (PA), and HDP], the incidence of perinatal outcomes [i.e., SGA, large-for-gestational-age (LGA), and preterm delivery (PTD)], the definition of outcomes, sample size of thin endometrial groups and thick endometrial groups, and other related information.

### Selection Criteria

Infertility women who underwent fresh cycles of IVF/ICSI treatment were included. Studies were included if these depicted the EMT of those women on ovulation triggering day and divided women into groups according to EMT. EMT, maximal distance from the endometrium–myometrium junction to the outer interfaces of the endometrium in the midsagittal plane of uterus, was measured by ultrasound examination. Moreover, the outcomes in those studies should be related to pregnancy, maternal or neonatal outcomes, such as live birth rate (LBR), CPR, SGA, and so on. The definition of outcomes should be specific.

We excluded studies, in patients with uterine pathology, such as fibroids, polyps, adenomyosis, and so on. Besides, donor oocytes, as a confounding factor, may affect generalizing the result as well, so studies with donor oocytes treatments were excluded. Those studies with no definition of outcomes or no EMT groups were also excluded.

### Types of Outcomes

The primary outcome was LBR, which was defined as at least one live born baby was delivered per cycles, irrespective of the duration of pregnancy (15). In addition, data were provided concerning CPR (defined as the number of clinical pregnancies that diagnosed by ultrasonographic visualization of one or more gestational sacs after positive human chorionic gonadotrophin (hCG) tests per cycles), implantation rate (IR, defined as the ratio of the number of gestational sacs to number of embryos transferred), early miscarriage rate (EMR, defined as pregnancy loss before 12 weeks following clinical pregnancy), and miscarriage rate (MR, calculated as the ratio of any pregnancy loss after clinical pregnancy to the number of clinical pregnancy) in terms of pregnancy outcomes as well (15–17).

Maternal outcomes were PA (defined as premature separation of the normally implanted placenta from the uterus) and PP (defined as placenta implants in the lower segment of the uterus and may cover part or all of the opening of the cervix). Moreover, HDP were also analyzed. Herein, HDP included gestational hypertension (defined as blood pressure ≥140/90 mmHg after 20 gestational weeks), preeclampsia (coexistence of gestational hypertension and one or both of the following new disorders: proteinuria; dysfunction of other maternal organs) and eclampsia (onset of hyperreflexia, seizures, or coma in a previously diagnosed preeclamptic women) (13, 14).

The neonatal outcomes of dichotomous variables included SGA (defined as birthweight <10th percentile of the average body weight at the same gestational week), large-for-gestational-age (LGA, defined as birthweight >90th percentile of the average body weight at the same gestational week), preterm delivery (PTD, defined as delivery before 37 weeks of gestational age) (11, 13). The continuous variable was birthweight (BW).

### Quality Assessment of Studies

The Newcastle-Ottawa Scale (NOS) that comprises eight items was employed to assess the quality of studies. It was used for evaluating the bias from selection, comparability and the outcomes assessment. One or two stars were awarded to each item and studies that met all criteria of the NOS would receive a maximum of nine stars. In the first item of NOS, representative cohorts were regarded as not restricted by diagnosis or by a type of ovarian stimulation protocol. Those cohort samples in fresh cycles that only received NC were also considered as unrepresentative. Moreover, studies adjusted for confounding factors (i.e., maternal age, body mass index, basal FSH, embryo score, chronic hypertension, previous pregnancy complications, and so on) by multivariable analysis or baseline data comparison were given a star or two stars in “comparability” item. Among them, age was the most important confounder that need to control. Besides, in “selection” and “outcome” items, information bias, such as the precision of measuring the EMT and evaluating the outcomes, were also taken into consideration. High-quality studies were considered as more than or equal 7 stars. Studies with medium quality had an total NOS score ≥5, but <7. Low quality studies had NOS score <5. Good quality and medium quality articles were included in the meta-analysis (18). Two investigators (ZL and LC) assessed risk of bias from each study *via* NOS independently. Disagreements between the two reviewers were settled by discussion and the third reviewer checked the accuracy of evaluation through view full-manuscript of those studies.

### Statistical Analysis

Given that most studies were retrospective cohort studies, we used odds ratios (OR) with 95%CI to measure dichotomous outcomes. Weighted mean difference (WMD) with 95%CI was used to analyze the association between EMT and BW. Results were combined for meta-analysis using Mantel–Haenszel fixed or random effects models which depended on heterogeneity. Q statistic and I^2^ statistics were used to evaluate the heterogeneity of studies. P <0.10 indicated the presence of heterogeneity, and I^2^ <50% indicated that the heterogeneity was acceptable, thus, a fixed‐effects model was used; otherwise, a random-effect model was used. Results were expressed as forest plots. Sensitivity analysis was conducted to examine heterogeneity and the robustness of the results. For meta-analysis of more than 10 articles, we also analyzed publication bias, which was assessed by funnel plot asymmetry and Egger’s test (P <0.05 considered as significant). When the publication bias existed, trim-fill adjustment method was used to assess the effect of this bias on outcomes. Statistics tests were calculated by the Review Manager software (version 5.3). Egger’s test and trim-fill analysis were analyzed by R (version 4.0.3).

## Results

### Literature Selection

There were 2,351 potential records by searching electronic database. After removing duplicates and screening titles and abstracts, 121 full-text articles related to our topic were retrieved for review. Of these, 82 records were excluded due to many reasons that are shown in **Figure 1**. Finally, 39 studies were eligible for further analysis. Considering that most studies select 7 and 14 mm as the threshold values for EMT grouping, we chose those thresholds as the cutoff values to explore the influence of thin (<7 mm) and thick (>14 mm) endometrium on pregnancy outcomes in the fresh cycles. Likewise, 7.5 mm was used as thin endometrial cut-off value for evaluating pregnancy complications and perinatal outcomes. Other studies (15 studies) that did not provide the above threshold information were not included for meta-analysis. Since most of studies were retrospective cohort studies, one prospective cross-sectional study was not suitable for meta-analysis, only for systematic review (19). In a study of fresh cycles, clomiphene (CC)-based minimal stimulation protocol was used, which was different from other controlled ovarian hyperstimulation (COH) protocols. Similarly, this study was only for systematic review as well (20). Therefore, 22 studies were included for final meta-analysis (5, 7, 11, 13, 16, 21–37).

### Description of Studies and Participants

Characteristics of included studies and patients are summarized in **Table 1**. The studies were published from 1991 to 2021. The articles used for meta-analysis were all observational studies, namely, retrospective and prospective cohort studies. Outcomes of most studies were LBR, CPR, IR, and MR. Herein, MR would be divided into two subgroups for meta-analysis, namely, EMR and MR. Only two articles described maternal (PP, PA, and HDP) and perinatal outcomes (SGA, BW, LGA, and PTD). Of these, BW was presented as mean with standard deviation (Mean ± SD).

**Table 1:** Characteristics of included references and participants.

Author (year)	Type of studies	Time-period	No. of patients	No. of cycles	Stimulation protocol	ART treatment	Type of cycles	Female age (Mean ± SD)	E2 on ovulation triggering day	EMT group (mm)	EMT measured day	Outcomes
Shakerian et al. (36)	Retrospective cohort study	10/2016-08/2019	NA	273	COH: GnRH-agonist/antagonist protocol.	IVF	Fresh cycles	36 (33–40)#	1,353.12 ± 754.13	<7, 7–14, >14.	hCG trigger	LBR, MR.
Simeonov et al. (37)	Retrospective cohort study	01/2009-12/2017	2343	5133	COH: GnRH-agonist/antagonist protocol.	IVF/ICSI	Fresh cycles	NA	NA	<7, >7	hCG trigger	LBR
Guo et al. (13)	Retrospective cohort study.	01/2017-12/2018	3157	NA	NC/Mild stimulation/COH: GnRH-agonist long/agonist short/antagonist protocol.	IVF/ICSI	Fresh cycles	31.52 ± 4.17	NA	<7.5, >7.5.	hCG trigger	PA/PP/HDP/SGA/LGA/PTD/BW.
Lv et al. (21)	Retrospective cohort study.	01/2013-12/2016	13909	15012	COH: GnRH-agonist long/agonist short/antagonist/minimal-stimulation/ultralong/other protocol.	IVF/ICSI	Fresh cycles	31.23 ± 5.29	3,289.68 + 1,915.22	<7, >7.	hCG trigger	LBR.
Tomicet al. (22)	Retrospective cohort study.	2010-2017	552	552	NC.	IVF	Fresh cycles	33.93 ± 3.41	250.14 ± 70.87	<7, 7–14, >14.	hCG trigger	CPR.
Nishihara et al. (20)	Retrospective cohort study	11/2018-03/2019	746	746	Clomiphene citrate-based minimal stimulation.	IVF/ICSI	Fresh cycles	38.1 ± 0.1*	NA	<7, >7.	hCG trigger	CPR.
Eftekhar et al. (5)	Retrospective cohort study.	05/2016-05/2018	1000	1000	COH: GnRH-agonist/antagonist protocol.	IVF/ICSI	Fresh cycles	NA	NA	<7, 7–14, >14.	hCG trigger	CPR
Ovayolu et al. (23)	Retrospective study.	2005-2013	359	359	COH: GnRH-agonist long/antagonist protocol.	IVF/ICSI	Fresh cycles	31.32 ± 4.01	2,299.56 ± 1,033.96	<7, 7–14, >14.	hCG trigger	LBR.
Song et al. (24)	Retrospective cohort study.	01/2013-12/2017	9511	4278	COH: short GnRH-agonist long protocol/prolonged protocol.	IVF/ICSI	Fresh cycles	28.93 ± 3.23	NA	<7, 7–14, >14.	hCG trigger	CPR/IR
Chan et al. (25)	Retrospective cohort study.	01/2012-12/2016	162	162	COH: GnRH-agonist/antagonist protocol.	IVF/ICSI	Fresh cycles	33.81 ± 3.65	1,886.10 ± 1,399.90	<7, 7–14, >14.	hCG trigger	LBR/CPR.
Holden et al. (26)	Retrospective cohort study.	05/2004-12/2012	6331	6180	COH: GnRH-agonist/antagonist protocol.	IVF/ICSI	Fresh cycles	35.6 (32.2–39.2) #	1,711 (1,012–2,691) #	<7, >7.	hCG trigger.	LBR
Oron et al. (11)	Retrospective cohort study.	01/2008-12/2014	864	5546	NC; COH: GnRH-agonist long/agonist short/antagonist protocol.	IVF/ICSI	Fresh cycles	32.49 ± 5.12	NA	<7.5, >7.5.	hCG trigger	PA/PP HDP/SGA/LGA/BW.
Ribeiro et al. (16)	Retrospective cohort study.	01/2010-12/2014	2827	3350	COH: GnRH-antagonist protocol.	IVF/ICSI	Fresh cycles	NA	NA	<7, >7.	hCG trigger	LBR/CPR/PTD/BW.
Wu et al. (27)	Retrospective cohort study.	01/2011-12/2013	2106	2106	COH: GnRH-antagonist protocol	IVF/ICSI	Fresh cycles	31.94 ± 3.71	2,771.20 ± 1,649.66	<7, 7–14, >14.	hCG trigger	CPR/IR
Zhao et al. (28)	Retrospective cohort study.	01/2009-05/2011	1933	3319	COH: HMG stimulation protocol.	IVF/ICSI	Fresh cycles	31.20 ± 4.60	3,489.70 ± 2,112.20	<7, 7–14, >14.	hCG trigger	CPR/IR
Aydin et al. (19)	Prospective cross-sectional study.	NA	593	593	COH: GnRH-agonist/antagonist protocol.	IVF/ICSI	Fresh cycles	26.86 ± 4.68	NA	<7, 7–14, >14.	hCG trigger	CPR/IR
Zhao et al. (29)	Retrospective cohort study.	01/2009-05/2011	1933	3319	COH: HMG stimulation protocol.	IVF/ICSI	Fresh cycles	31.18 ± 4.62	3,489.62 ± 2,112.21	<7, 7–14, >14.	hCG trigger	CPR/IR
Chen et al. (30)	Retrospective cohort study.	01/2003-12/2008	2896	2896	COH: GnRH-agonist long protocol.	IVF/ICSI	Fresh cycles	31.00 ± 3.90	2 107.30 ± 1,596.10	<7, 7–14, >14.	hCG trigger	CPR
Okohue et al. (31)	Prospective study.	05/2005-04/2006	251	251	COH: GnRH-agonist long protocol.	IVF/ICSI	Fresh cycles	30.58 ± 3.35	NA	<7, 7–14, >14.	hCG trigger	CPR
Al-Ghamdi et al. (32)	Retrospective cohort study.	01/2003-12/2005	2464	2464	COH: GnRH-agonist long/agonist short protocol.	IVF/ICSI	Fresh cycles	30.83 ± 5.45	NA	<7, 7–14, >14.	hCG trigger	CPR
Richter et al. (33)	Retrospective cohort study	01/2002-12/2005	1294	1294	COH	IVF/ICSI	Fresh cycles	33.67 ± 3.47	2,553.67 ± 991.13	<7, 7–14, >14.	hCG trigger	LBR/CPR
Yoeli et al. (34)	Prospective study.	1998-2000	783	1218	COH: GnRH-agonist long/agonist short protocol.	IVF/ICSI	Fresh cycles	32.86 ± 4.70	1,329.78 ± 1,053.67	7–14, >14.	hCG trigger	CPR/IR
Weissman et al. (7)	Retrospective cohort study.	1994-1995	NA	717	COH: GnRH-agonist long protocol.	IVF/ICSI	Fresh cycles	NA	NA	7–14, >14.	hCG trigger	CPR/IR
Noyes et al. (35)	Prospective study.	10/1991-06/1992	477	516	COH: GnRH-agonist/Only Gn stimulation/CC+ Gn stimulation protocol.	IVF	Fresh cycles	35.90 ± 4.20	1,465.00 ± 798.00	<7, 7–14, >14.	hCG trigger	CPR/IR

ART, artificial reproductive technology; IVF, in vitro fertilization; ICSI, intracytoplasmic sperm injection; E2, estradiol; COH, controlled ovarian hyperstimulation; GnRH, gonadotropin releasing hormone; Gn, gonadotrophin; CC, Clomiphene citrate; NC, natural cycle; EMT, endometrial thickness; hCG, human chorionic gonadotrophin; PA, placenta abruption; PP, placenta previa; HDP, hypertensive disorders of pregnancy; SGA, small-for-gestational-age; LGA, large-for-gestational-age; PTD, preterm delivery; BW, birthweight; LBR, live birth rate; CPR, clinical pregnancy rate; MR, miscarriage rate; IR, implantation rate; NA, Not applicable. 2) E2 (pg/ml): data are presented as mean + SD. 3) *: mean± SEM; #: median (interquartile range).

All women underwent the fresh cycles of IVF/ICSI treatment. Women were divided into three groups depending on EMT when analyzed pregnancy outcomes (Thin endometrium/decreased EMT group: EMT <7mm; Medium endometrium group: EMT 7–14 mm; Thick endometrium/increased EMT group: EMT >14 mm). The effect of thin endometrium on obstetric complications and perinatal outcomes was evaluated in endometrial cut-off value of < 7.5cm versus >7.5cm. The total number of reported patients and cycles that were related to LBR was about 27,225 and 31,763 respectively. The number of patients enrolled for maternal and perinatal outcomes was 4,021. The mean of female age was approximately between 29 and 36 years. COH, such as GnRH-agonist long or short protocols and GnRH-antagonist protocols, were used in patients. On hCG triggering day, the mean of E2 level of these patients was about from 1,329.78 pg/ml to 3,489.62 pg/ml.

### Quality of Studies

The quality of studies based on NOS is shown in **Supplementary Table 2**. Qualities of 14 studies were good level, and 8 studies were medium level. Therefore, all 22 studies were included in analysis.

### Live Birth Rate

**Figure 2:**
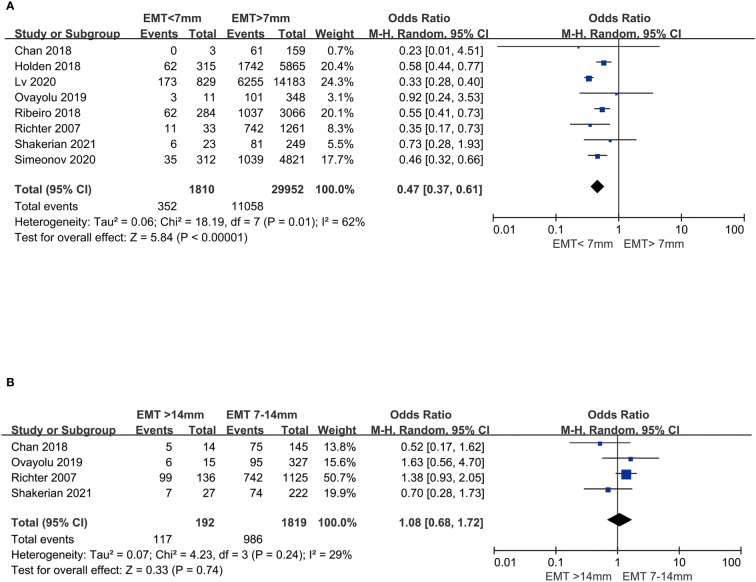
Comparison of LBR between EMT groups in fresh cycles. **(A)** Comparison between thin endometrium group and non-thin endometrium group. **(B)** Comparison between thick endometrium group and medium endometrium. LBR, Live birth rate; EMT, Endometrial thickness.

Women with thin endometrium (EMT <7 mm) had a significantly lower LBR compared to those women with EMT >7 mm in fresh cycles (OR 0.47, 95%CI: 0.37, 0.61, P <0.00001) (**Figure 2A**). However, significant heterogeneity was observed in this result (I^2^ = 62%). Hence, sensitivity analysis was conducted to detect the stability of result by removing each study and re-analyzing the remaining studies, which did not change the direction of the effect. When one study (21) was excluded, the substantial heterogeneity was decreased (I^2^ declined from 62 to 0%). Women with thick endometrium (EMT >14 mm), had no significant higher LBR than those with medium EMT (7–14 mm) in fresh cycles (OR 1.08, 95%CI: 0.68, 1.72, P = 0.74, low heterogeneity: I^2^ = 29%) (**Figure 2B**).

### Clinical Pregnancy Rate

**Figure 3:**
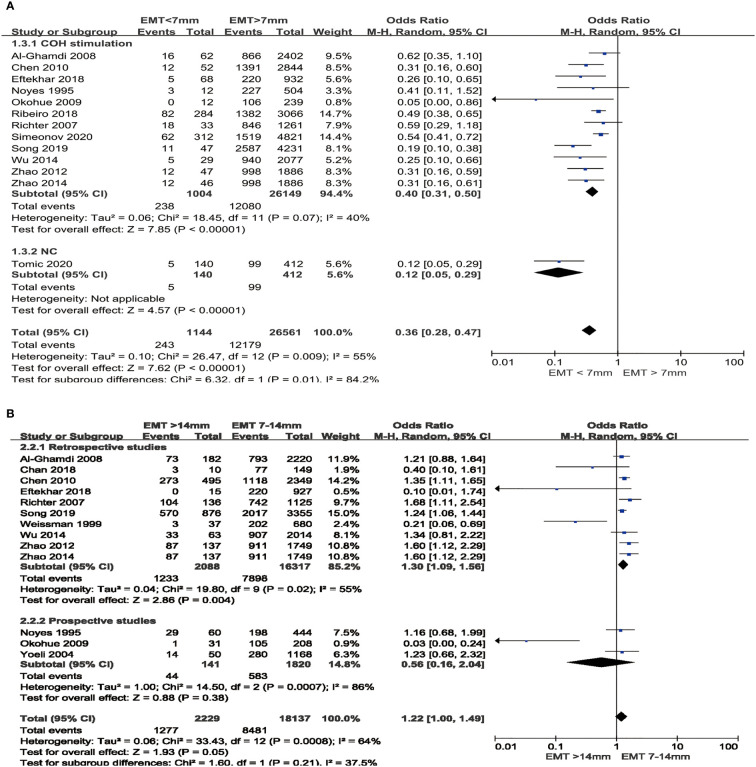
Comparison of CPR between EMT groups in fresh cycles. **(A)** Comparison between thin endometrium group and non-thin endometrium group. **(B)** Comparison between thick endometrium group and medium endometrium. CPR, Clinical pregnancy rate; COH, Controlled ovarian hyperstimulation; NC, Natural cycles.

Twelve studies that reported CPR of women with thin endometrium in fresh cycles are shown in **Figure 3**. Subgroup analysis was conducted according to whether women underwent COH protocols. In fresh cycles, lower CPR of decreased EMT group was observed both in COH stimulation group (OR 0.40; 95%CI: 0.31, 0.50, P <0.00001, low heterogeneity: I^2^ = 40%) and in NC group. Since the analysis in COH stimulation group included more than 10 studies, funnel plot was presented (**Supplementary Figure 1**) and Egger’ test (intercept = −0.4333, t = −2.99, P = 0.0135) was estimated. However, those results indicated the presence of publication bias, so the trim-fill adjustment method was analyzed. After adjustment, the ORs changed from 0.40 to 0.48, and the significant level did not change, which suggested that the existing results were not affected by publishing bias.

Thirteen studies that reported CPR of women with thick endometrium in fresh cycles are also shown in **Figure 3**. We performed subgroup analysis as well according to the study types. When only retrospective studies included for meta-analysis, result showed that women with thick endometrium had higher chances to conceive (OR 1.30; 95%CI: 1.09, 1.56, P = 0.004). When, notwithstanding, prospective studies were also included for analyzing, it seemed that there was no significant difference in CPR between thick endometrium and medium endometrium group in fresh cycles (OR 1.22; 95%CI: 1.00, 1.49, P = 0.05). It should be noted that substantial heterogeneity existed among all studies (I^2^ = 64%), so we performed sensitivity analysis. When two (7, 31) of the studies was removed separately, the heterogeneity decreased (I^2^ declined from 64 to 54%, or to 45% respectively) and the result changed (OR 1.29, 95%CI: 1.09, 1.54; OR 1.29, 95%CI: 1.11, 1.51 respectively). This analysis indicated that the result was not robust to some extent. Similarly, there were 10 studies in retrospective studies subgroup, so publication bias also estimated *via* funnel plot and Egger’s test (**Supplementary Figure 2**). No publication bias was presented after assessed by Egger’s test (intercept = 0.4283, t = −1.61, P = 0.1471).

### Implantation Rate

**Figure 4:**
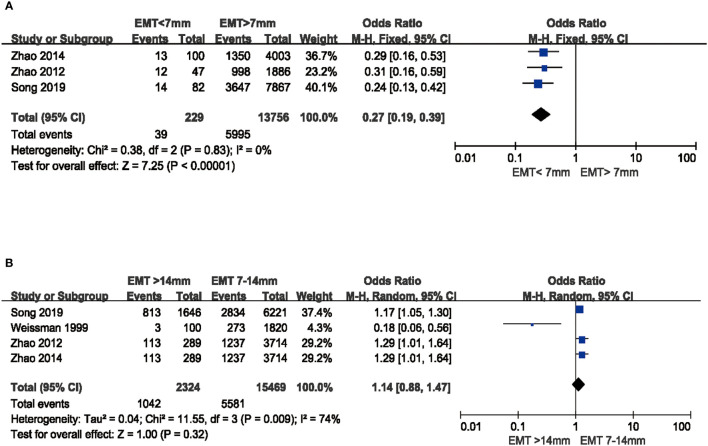
Comparison of IR between EMT groups in fresh cycles. **(A)** Comparison between thin endometrium group and non-thin endometrium group. **(B)** Comparison between thick endometrium group and medium endometrium. IR, Implantation rate.

Similar to the results of LBR and CPR, thin endometrial patients had lower IR than those with EMT >7 mm as well (OR 0.27, 95%CI: 0.19, 0.39, P <0.00001, no heterogeneity: I^2^ = 0) (**Figure 4A**). However, there was no significant difference among patients with thick endometrium compared to medium endometrium group (OR 1.14, 95%CI: 0.88, 1.47, P = 0.32), though the substantial heterogeneity (I^2^ = 74%) existed (**Figure 4B**).

### Miscarriage Rate

**Figure 5:**
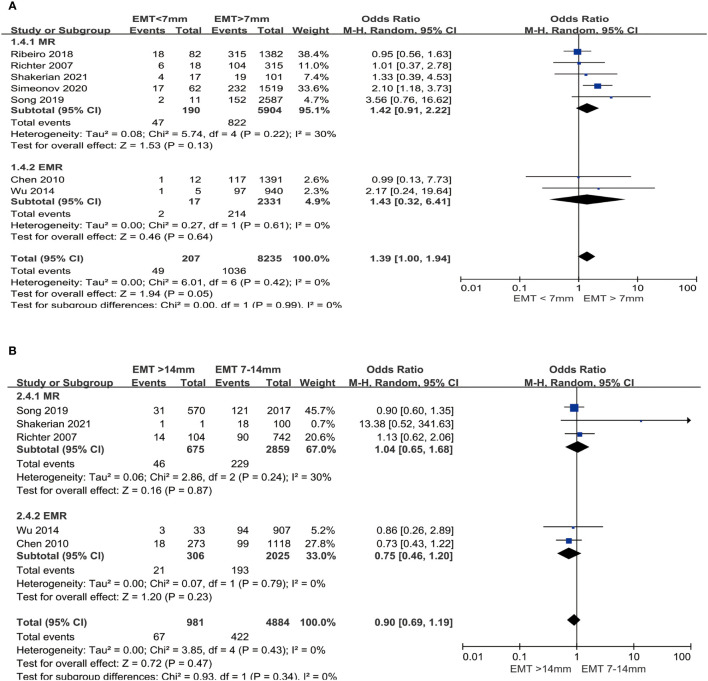
Comparison of MR between EMT groups in fresh cycles. **(A)** Comparison between thin endometrium group and non-thin endometrium group. **(B)** Comparison between thick endometrium group and medium endometrium. MR, Miscarriage rate; EMR, Early miscarriage rate.

In a subgroup analysis, no significant difference was observed in EMR (OR: 1.43, 95%CI: 0.32, 6.41, P = 0.64, no heterogeneity: I^2^ = 0%) and MR (OR 1.42; 95%CI: 0.91, 2.22, P = 0.13, low heterogeneity: I^2^ = 30%) in women with thin endometrium than those with EMT >7 mm in fresh cycles (**Figure 5A**). In an analysis about the effect of thick endometrium on EMR or MR, there was also no significant difference comparing thick endometrium groups to medium endometrium groups (MR: OR 1.04, 95%CI: 0.65, 1.68, P = 0.87, low heterogeneity I^2^ = 30%; EMR: OR 0.75, 95%CI: 0.46, 1.20, P = 0.23, no heterogeneity I^2^ = 0%) (**Figure 5B**).

### Systematic Review

A prospective cross-sectional study from Aydin et al. showed that there were significantly lower CPR and IR in thin endometrium group in fresh cycles (P <0.05, P <0.05, respectively), which also corroborated our results of meta-analysis. Furthermore, a study from Nishihara et al. also showed that CPR was significantly decreased in women with thin endometrium in fresh cycles of CC-based stimulation (P <0.05).

### Maternal and Perinatal Outcomes

**Figure 6:**
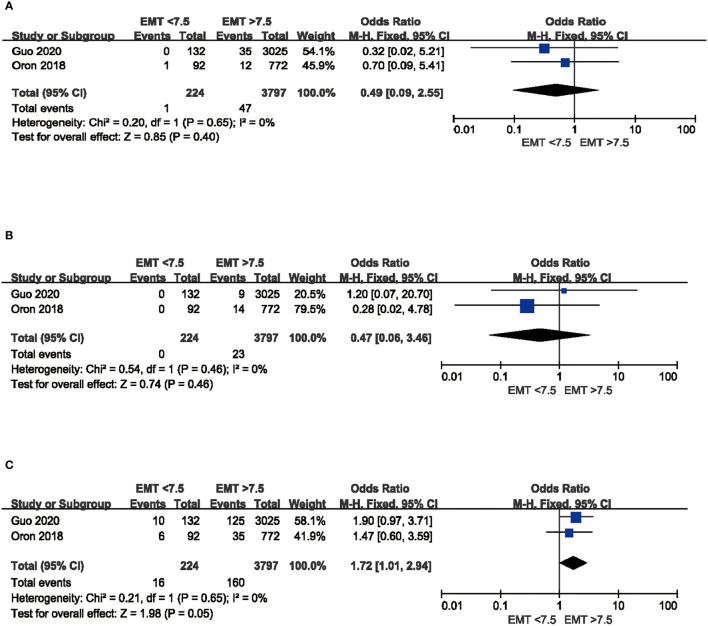
Comparison of maternal outcomes between EMT <7.5 mm and EMT >7.5 mm in fresh cycles. **(A)** Comparison of PP between thin endometrium group and non-thin endometrium group. **(B)** Comparison of PA between thin endometrium group and non-thin endometrium group. **(C)** Comparison of HDP between thin endometrium group and non-thin endometrium group. PP, Placenta previa; PA, Placenta abruption; HDP, hypertensive disorders of pregnancy.

With respect to obstetric outcomes, as shown in **Figure 6**, thin endometrium (EMT <7.5 mm) had no effect on placenta previa (OR 0.49, 95%CI: 0.09, 2.55, P = 0.40, no heterogeneity I^2^ = 0%) and placenta abruption (OR 0.47, 95%CI: 0.06, 3.46, P = 0.46, no heterogeneity I^2^ = 0%). Incidence of hypertensive disorders of pregnancy was increased in women with thin endometrium, but there was no significant difference (OR 1.72, 95%CI: 1.01, 2.94, P = 0.05, no heterogeneity I^2^ = 0%).

**Figure 7:**
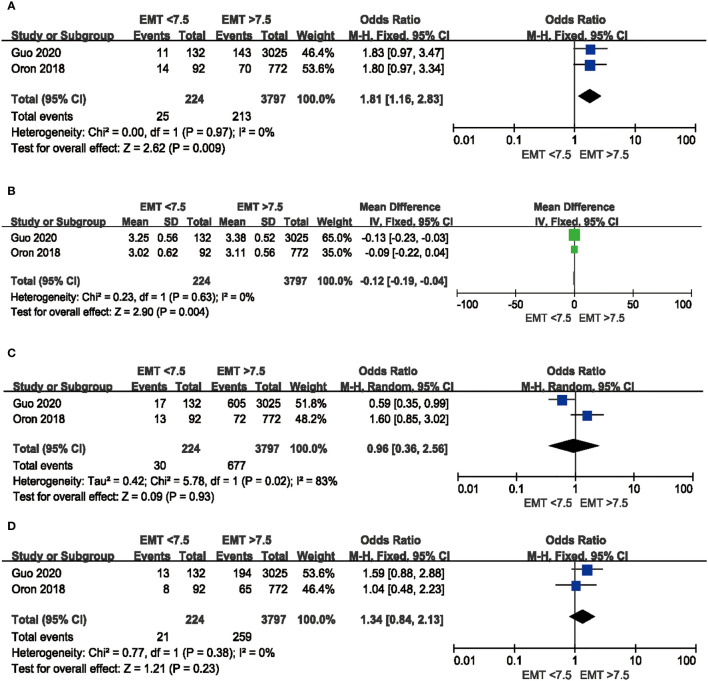
Comparison of perinatal outcomes between EMT <7.5 mm and EMT >7.5 mm in fresh cycles. **(A)** Comparison of SGA between thin endometrium group and non-thin endometrium group. **(B)** Comparison of BW between thin endometrium group and non-thin endometrium group. **(C)** Comparison of LGA between thin endometrium group and non-thin endometrium group. **(D)** Comparison of PTD between thin endometrium group and non-thin endometrium group. SGA, Small-for-gestational-age; BW, Birthweight; LGA, Large-for-gestational-age; PTD, Preterm delivery.

Besides, perinatal outcomes, such as small-for-gestational-age, large-for-gestational-age, and preterm delivery are shown in **Figure 7**. A higher incidence of SGA was observed in infants from decreased EMT group (OR 1.81; 95%CI: 1.16, 2.83; P = 0.009, no heterogeneity I^2^ = 0%) and babies had significantly lower BW from women with thin endometrium (WMD: −0.12 kg, 95%CI: −0.19, −0.04, P = 0.004, no heterogeneity I^2^ = 0%). No significant difference was observed in the incidence of LGA (OR 0.96, 95%CI: 0.36, 2.56, P = 0.93, high heterogeneity: I^2^ = 83%) and PTD (OR 1.34, 95%CI: 0.84, 2.13, P = 0.23, no heterogeneity I^2^ = 0%) neonates in the thin endometrium group.

## Discussion

In this review, we analyzed the effect of EMT on pregnancy, maternal, and perinatal outcomes in women after fresh cycles of IVF/ICSI. Because there was no consensus on the definition of thin or thick endometrium, we selected cutoffs of thin or thick endometrium reported in most studies for our meta-analysis, such as 7 and 14 mm in fresh cycles. Similarly, as the number of studies related to maternal and perinatal outcomes was not enough and the cutoffs of thin endometrium in these studies also have not reached an agreement, 7.5 mm that reported in most studies was selected for analyzing.

We found that LBR, CPR, and IR were lower in patients with thin endometrium, which were consistence with previous studies (4, 22, 38). The underlying reason might not only be related to high oxygen levels in basal layer of endometrium, but also relevant to abnormal transcriptional changes in thin endometrium (39–41). For instance, a recent study revealed that differentially expressed genes and microRNAs, which were enriched in angiogenesis, cell growth regulation, and Wnt signaling pathway, were detected in the mid-secretary phase of thin endometrium compared to adjacent normal endometrial cells (41). Moreover, our results showed that though a thin endometrium had no effect on MR, but had higher chance of early miscarriage. Although the reason behind this phenomenon is unclear, we speculated that decreased EMT had detrimental effect on decidualized endometrium, so this disrupt might contribute to some implanted embryos destined to miscarry before 12 weeks of gestation (42).

In terms of thick endometrium, there was no significant association between increased EMT and LBR, CPR, IR and MR. It should be noted that no significant difference was demonstrated between CPR in thick EMT and medium EMT group due to the substantial heterogeneity that existed among the studies. From the above results, it is clear that thick endometrium does not increase MR nor decrease CPR. Thus, thick endometrium does not have adverse effects on IVF outcomes, which is also supported by previous studies (4, 34, 43).

Apart from pregnancy outcomes, the obstetric complications (like HDP) and the perinatal outcomes (such as BW and SGA), were revealed to be influenced by EMT. Of these, the thickness of the endometrium has a negative relationship with the incidence of HDP or SGA and a positive correlation with BW, which were in accordance with previous studies (13, 14, 44). Notwithstanding, the number included in the studies is still insufficient, so it cannot make a firm conclusion and demands to be confirmed in a large sample prospective cohort study. Normal placental function and fetal development are both relied on the intrauterine environment (45). It is believed that the development of HDP and fetal growth restriction result from the failure of transformation of uterine spiral arteries into large vessels (45, 46). We speculated there was abnormal uterine artery blood flow in thin endometrium, as a consequence that intrauterine environment could not be maintained and the risk of HDP or SGA also increased. Moreover, a study revealed that thin endometrium appears to be associated with an aberrantly activated inflammatory environment (40). Thus, the increased immunological factors in thin endometrium may also impair placentation and contribute to the occurrence of SGA or preeclampsia (47, 48). However, the underlying mechanism for this phenomenon is still unclear and needs to be elucidated.

Our study provided evidence that thin endometrium not only dampened the pregnancy outcomes following in IVF/ICSI, but also suppressed the fetal development, namely, increased the risk of SGA and decreased the BW of the fetus. The incidence of HDP arose, suggesting thin endometrium might also contribute to abnormal placental functions. However, because of the small number of included studies, the conclusion needs to be drawn with caution. In general, clinicians need to inform patients of possible obstetric complications caused by thin endometrium after IVF/ICSI and encourage patients to actively cooperate with prenatal examinations and receive more perinatal care after conceiving.

Previous studies showed that thick endometrium had negative effect on IVF/ICSI pregnancy (7). Our results suggested that increased EMT did not adversely affect the pregnancy outcome. This phenomenon might be helpful for clinicians to make decisions about embryo transplantation when they encounter thicker endometrium.

This was, to the best of our knowledge, the first meta-analysis that not only explored the role of thick endometrium on pregnancy outcomes but also analyzed the effect of EMT on obstetric complications and perinatal outcomes after IVF/ICSI. Understanding these influences may enable evidence-based support to be provided.

There are also some limitations in this study. Firstly, substantial heterogeneity among studies existed in some analysis, such as when analyzing the effect of thin endometrium on LBR or CPR, and the influence of thick endometrium on CPR or IR. Secondly, many of the included studies were retrospective studies and this study type is relevant to an inevitable risk of bias. Thirdly, as the different sonographers and equipment cause, the measurements of EMT are inherent with inter- and intra-variability, which might also bring some bias. Additionally, the definition of thin endometrium has not reached an agreement (38). In our study, 7 mm was chosen as the cutoff value for thin EMT as most studies reported, and thus, this selection method might ignore the influence of other thresholds on outcomes. Fourthly, the cause of thin endometrium is unclear in studies and it is possible that scarred thin endometrium, such as following curettage, entails a poorer prognosis than “natively” thin endometrium, which might also affect the results (49). Lastly, because the number of studies related to maternal and perinatal outcomes is insufficient and the inclusion of any studies relating to impaired fetal growth did not refer to long term neuro development, more well-conducted prospective studies are required.

In conclusion, our study indicated that thin endometrium had an adverse effect on LBR, CPR, IR and BW of infants, and increased the incidence of HDP in women and SGA of babies. However, it had little effect on MR, PA, and PP of patients, or on LGA and PTD among infants. More observational studies with large sample sizes and long-term follow-up or more randomized trials with preset protocols need to investigate the significance of the EMT on maternal or perinatal outcomes following in IVF/ICSI. The thick endometrium made no significant difference to pregnancy outcomes in fresh cycles.

## Data Availability Statement

The original contributions presented in the study are included in the article/**Supplementary Material**. Further inquiries can be directed to the corresponding authors.

## Author Contributions

ZL and CL contributed to the design of study. ZL, LC, and LS performed studies search and data collection. ZL and CL drafted the manuscript, which was revised by KQ, CS, and HZ. All authors contributed to the article and approved the submitted version.

## Conflict of Interest

The authors declare that the research was conducted in the absence of any commercial or financial relationships that could be construed as a potential conflict of interest.

## Publisher’s Note

All claims expressed in this article are solely those of the authors and do not necessarily represent those of their affiliated organizations, or those of the publisher, the editors and the reviewers. Any product that may be evaluated in this article, or claim that may be made by its manufacturer, is not guaranteed or endorsed by the publisher.
