# Supplementary material for: The Effect of Endometrial Thickness on Pregnancy, Maternal, and Perinatal Outcomes of Women in Fresh Cycles After IVF/ICSI: A Systematic Review and Meta-Analysis
Source: Front Endocrinol (Lausanne). 2022 Feb 11;12:814648. doi: 10.3389/fendo.2021.814648 (PMC8874279; doi:10.3389/fendo.2021.814648)

Supplementary Table 1 Search strategy for databases

Database	Search strategy	No. of references
Pubmed	((in vitro fertilization) OR (intracytoplasmic sperm injection) OR (artificial reproductive technology)) AND ((endometrial thickness) OR (endometrial sonographic parameters) OR (endometrial characters) OR (endometrial receptive)) AND ((live birth rate) OR (pregnancy outcomes) OR (neonatal outcomes) OR (maternal outcomes) OR (obstetric outcomes) OR (treatment outcomes))	538
Cochrane library	((in vitro fertilization) OR (intracytoplasmic sperm injection) OR (artificial reproductive technology)) AND ((endometrial thickness) OR (endometrial sonographic parameters) OR (endometrial characters) OR (endometrial receptive)) AND ((live birth rate) OR (pregnancy outcomes) OR (neonatal outcomes) OR (maternal outcomes) OR (obstetric outcomes) OR (treatment outcomes)) in Title Abstract Keyword - (Word variations have been searched)	293
Embase	(in AND vitro AND ('fertilization'/exp OR fertilization) OR (intracytoplasmic AND ('sperm'/exp OR sperm) AND ('injection'/exp OR injection)) OR (artificial AND reproductive AND ('technology'/exp OR technology))) AND (endometrial AND ('thickness'/exp OR thickness) OR (endometrial AND sonographic AND ('parameters'/exp OR parameters)) OR (endometrial AND characters) OR (endometrial AND receptive)) AND (live AND ('birth'/exp OR birth) AND rate OR (('pregnancy'/exp OR pregnancy) AND ('outcomes'/exp OR outcomes)) OR (neonatal AND ('outcomes'/exp OR outcomes)) OR (('maternal'/exp OR maternal) AND ('outcomes'/exp OR outcomes)) OR (obstetric AND ('outcomes'/exp OR outcomes)) OR (('treatment'/exp OR treatment) AND ('outcomes'/exp OR outcomes)))	685
Web of science	(((in vitro fertilization) OR (intracytoplasmic sperm injection) OR (artificial reproductive technology)) AND ((endometrial thickness) OR (endometrial sonographic parameters) OR (endometrial characters) OR (endometrial receptive)) AND ((live birth rate) OR (pregnancy outcomes) OR (neonatal outcomes) OR (maternal outcomes) OR (obstetric outcomes) OR (treatment outcomes)))	836
Total	/	2351

Supplementary Table 2 Quality assessment of included studies based on Newcastle-Ottawa Scale.

Study	Selection				Comparability	Outcome			Score
	Representativeness of the exposed cohort	Selection of the non-exposed cohort	Ascertainment of the exposure	Demonstration that outcomes of interest was not present at start of study	Comparability of cohorts on the basis of the design or analysis	Assessment of outcome	Was follow-up long enough for outcomes to occur	Adequacy of follow-up of cohorts	
Shakerian 2021	*	*	*		*	*	*	*	7
Simeonov 2020	*	*	*		*	*	*	*	7
Guo 2020	*	*	*		**	*	*	*	8
Lv 2020	*	*	*		*	*	*	*	7
Tomic 2020		*	*		*	*	*	*	6
Nishihara 2020		*	*		*	*		*	5
Eftekhar 2019	*	*	*			*	*	*	6
Ovayolu 2019		*	*		*	*	*	*	6
Song 2019		*	*		*	*	*	*	6
Chan 2018	*	*	*		*	*	*	*	7
Holden 2018	*	*	*		*	*	*	*	7
Oron 2018	*	*	*		*	*	*	*	8
Ribeiro 2018		*	*		**	*	*	*	7
Wu 2014		*	*		**	*	*	*	7
Zhao 2014	*	*	*		*	*	*	*	7
Aydin 2013		*	*	*	**	*			6
Zhao 2012	*	*	*			*	*	*	6

Chen 2010		*	*		*	*	*	*	6
Okohue 2009		*	*	*	*	*		*	6
AL-Ghamdi 2008	*	*	*		*	*	*	*	7
Richter 2007	*	*	*		*	*	*	*	7
Yoeli 2004		*	*	*	**	*	*	*	8
Weissman 1999		*	*		*	*	*	*	6
Noyes 1995	*	*	*	*	*	*		*	7

Supplementary Fig. 1 Funnel plot of studies related to the effect of thin endometrium on CPR.

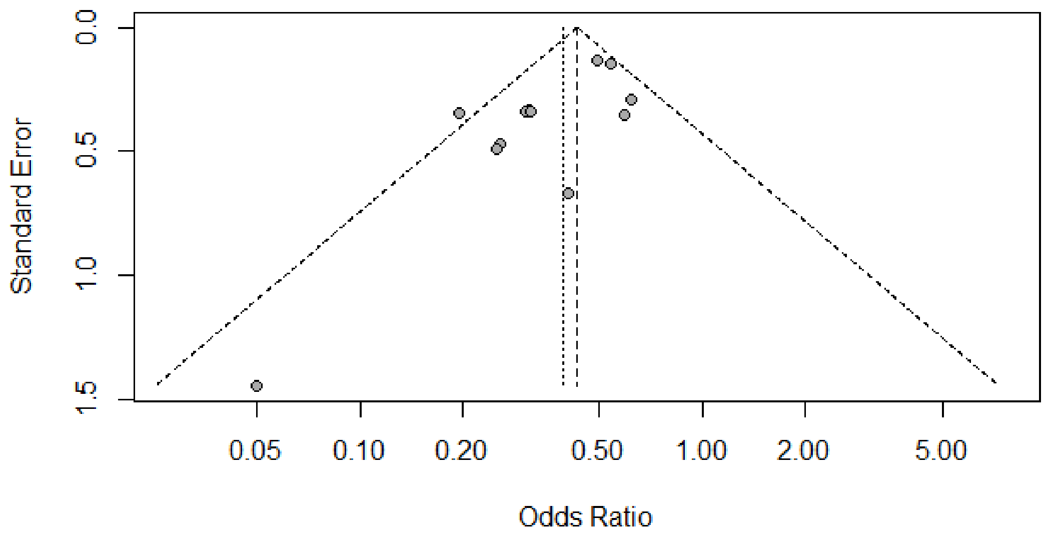

Supplementary Fig. 2 Funnel plot of studies related to the effect of thick endometrium on CPR.

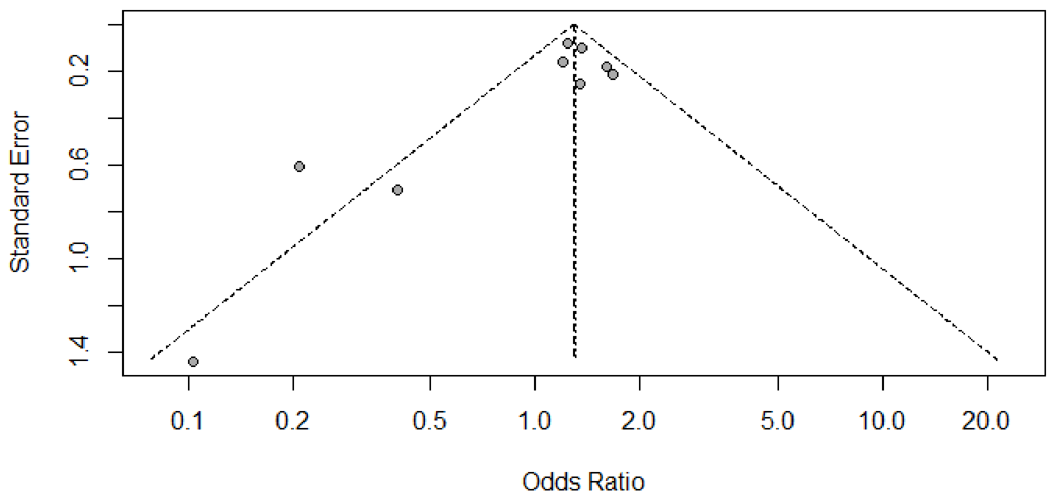

Supplement: Supplementary file 1 [file DataSheet_1.pdf]
